# Supplementary material for: Inhibition of transcriptional regulation of detoxification genes contributes to insecticide resistance management in Spodoptera exigua
Source: Commun Biol. 2025 Jan 27;8:128. doi: 10.1038/s42003-025-07560-8 (PMC11772755; doi:10.1038/s42003-025-07560-8)
Supplement: Supplementary file 1 — Supplementary information [file 42003_2025_7560_MOESM1_ESM.pdf]

Inhibition of transcriptional regulation of detoxification genes contributes to insecticide resistance management in *Spodoptera exigua*

Bo Hu[1,2], Yuping Deng[1,2], Tao Lu[1,2], Miaomiao Ren[3], Kuitun Liu[4], Cong Rao[4], Hailiang Guo[4], Jianya Su[4]*

[1] Jiangsu Key Laboratory of Sericultural and Animal Biotechnology, School of Biotechnology, Jiangsu University of Science and Technology, Zhenjiang 212100, China.

[2] Key Laboratory of Silkworm and Mulberry Genetic Improvement, Ministry of Agriculture and Rural Affairs, Sericultural Scientific Research Center, Chinese Academy of Agricultural Sciences, Zhenjiang 212100, China.

[3] College of Environment and Chemical Engineering, Jiangsu University of Science and Technology, Zhenjiang 212100, China.

[4] Key Laboratory of Integrated Management of Crop Diseases and Pests (Ministry of Education), College of Plant Protection, Nanjing Agricultural University, Nanjing 210095, China

*Corresponding author:

Jianya Su, PhD, College of Plant Protection, Nanjing Agricultural University, Nanjing 210095, China. Email: sjy@njau.edu.cn.

This file includes:
Table: S1-S5
Figure: S1-S9

Supplementary Table 1 Primers used for amplification of ABCBs and CarEs.

Gene	Primer sequences (5'-3')
ABCB2-F	TCATGCCCTTCTCAGTAGCAGCCTC
ABCB2-R	CGTCTAGCGCAGAAGTGGCTTCATC
CarE1-F	ATGAAATATGGGATTTGTTCGAAT
CarE1-R	CTATACACATTCACAAGTGGCAAAT
CarE2-F	ATGAACTACATGTTCGCGCTCCTG
CarE2-R	TTACATCACATTCACTTTACTGTG
CarE3-F	ATGGTGGAAGTTAAAGTGGAACAA
CarE3-R	TCAATATTCCACTCCAGCGTAATC

Supplementary Table 2 Primers used in quantitative real-time PCR of genes

Name	Sense primer	Anti-sense primer	Amplicon (bp)	qRT-PCR efficiency (%)
CYP321A16	GCCGTCGGTATAGGTCAGTT	CAATGCTTTCCACTCCTCGG	118	100.6
CYP321A9	CAGAAAGGAACCAGCCATCG	CCTTCGACGCTTTCCATACC	135	103.4
CYP321A8	AAACAACCCCAAGACGCATG	CCAATGCCAAAGATAGCCCC	90	105.6
CYP321B1	TGGAGCCTACAGACGAGTTG	TCCAAAAGTTCCGGCAGTTG	86	91.99
CYP9A27	ATGACGATAAACCCAATG	CAAAGCCAGCGATAAAGA	141	95.78
CYP9A11	ATCTTACAACTCGCTACGC	GCTGCTGTCCTACCCATTA	112	94.56
Maf	GTCGTCGCACTCTGAAGAAC	CCTGCATCAACTCCATGTCG	120	98.49
CncC	GGGACAGGAAACAGAGGACA	ATTGTTGGGAGGATAGCGGT	144	98.78
GSTe2	TCGTCGACCAATGCATGTTC	CGGCAGCTACGTACTTGTTC	177	90.88
GSTe6	GTCAACGTAAAGGCTGGTGA	CGTCAGAATTGCATGGCTGT	120	93.32
GSTe14	TGAGTCACTTGGCTACCCTC	ACGGCGTTGCTTTGAAATCT	96	93.99
GSTo2	TCTATCACCGATCCACACCG	AGGGCCAGATCATGTAGTCG	179	99.21
ABCB1	GGGGCTACTGGTCAAAGGAT	CTCGCCACTCAAAGATCAGC	91	100.02
ABCB2	ACAGTCTCAGTACACAGGGC	ACAGCCTGAGGACCCTACTA	167	98.34
ABCC1	TGCGCCATCATAGGATCTGT	GGGCCCCGAGAGATAGATTC	102	97.46
ABCC2	CCCAGATCCTGCATTGAACG	AGCTCGCATAGTCATCACGA	139	98.01
ABCC3	CGTTTACTGCCGAGCTCTTC	TTCCGGATGCCCCTTTAAGT	89	99.13
CarE01	ACGTCAAAGGGTCCGGTAAT	CCATTGCAGCTACTTGGTCT	168	98.42
CarE02	TTCTTCAGTACCTCCCCTGC	TGTCACCAGCCCCAACATAT	171	95.47
CarE03	GACAGTGACCGGAGATGGAA	TTGCTTTTCTGACGCCTTCC	125	96.78
CarE04	AAGATAGAGCTGCGTGGTGT	TGGTTTTGGAGGTACAGGCT	96	97.06
CarE05	GGACTCACACGGAAAACCAC	TCCGTATCTGGCGAAGTTGT	157	99.82
GAPDH	CTGAGGAACAGGTCGTGTCATC	GATCGATAACGCGGTTGGAGTA	150	99.67
β-Actin	AAGCCTTCGATGCCACCGGGTA	TTCGGGCGTGTTTAGTGGAGGC	170	99.73

Supplementary Table 3 The primers used for cloning 5′-flanking regions

Primers	Primer sequences (5' - 3')
PCYP321A8-1	ACTGGAAGTCTCCTGATAGAACCTGCT
PCYP321A8-2	ACCTCAAAAAGAGCACGCTGCGCTATG
PCYP321A8-3	AATTTGCAGCTCAGAAACAGTATACTGT
PCYP321A8-4	CAGCCGTTCTAATTTTGCTGAGTCATGA
PCYP321A8-5	TACTGAATACGAGATAACTCTCTGAACC
PCYP321A8-6	CACGACTGAAAGGTTAAGGTTAAGGTTG
PCYP321A8-7	GATAAGAGTTATCTAACATCGACTAATG
PCYP321A8-8	CAGCTTTACCTCATGGTGACAGGCAGAA

CYP321A8-DNA-F	GGTCAATGAATTGTGTAACATCATC
CYP321A8-DNA-R	GCCTACTGCTACTAAACTCAAAGGT
Adaptor Primer1	GTAATACGACTCATCATAGGGC
Nested Adaptor Primer2	ACTATAGGGCACGCGTGGT
PCYP321A9-1	CGTAGAAGGTAACACTACGTTTCCTCCA
PCYP321A9-2	TGTACAGCTTTCCGAAGATCTCGAACAG
CYP321A9-DNA-F	GTAACTTGGTAGGTACATTGCTACG
CYP321A9-DNA-R	CGTTGTGTATTTTTCTGAAGCGTGAG
PCYP321A16-1	CTGACCTATACCGACGGCTGGTTCATG
PCYP321A16-2	GATCGCATCAGCTTCCATCTTGGACCA
PCYP321A16-3	AACTGACTTCAATTGACTTCACGAGCAG
PCYP321A16-4	AATTGCGCGCCGTACTTATACTCAGCTG
CYP321A16-DNA-F	TGTCTGCTCATTTCACATTTCGTAC
CYP321A16-DNA-R	CGTTAATTTTTTGTCGTGAGTTCGTA
PCYP321B1-1	ATGGTCTTTCTCCGATCATGTACTCCCA
PCYP321B1-2	TGACGTAGAGACCATGGGAGTCGAACGA
CYP321B1-DNA-F	CGAAACCTTTTCCATGACTCGATGA
CYP321B1-DNA-R	GTTTGGTTGTCTGAACTTGTCTAAG
PCYP9A11-1	TGCCATGTTTACTGAATGCAGAGTGCAT
PCYP9A11-2	GAACATCATAGGCGAAGTGCTCTCGTAG
CYP9A11-DNA-F	ATCAAAGATAAAGCTGTTAAGACAC
CYP9A11-DNA-R	TGCCATGTTTACTGAATGCAGAGTG
PCYP9A27-1	CAACCGCACAGAGAATCCATATTATCAT
PCYP9A27-2	CATGACTGAGGTCATGTTGCCCAGCAGT
CYP9A27-DNA-F	CAGGTATGCACGCAGGGAATATGCA
CYP9A27-DNA-R	CAACCGCACAGAGAATCCATATTAT
GSTe6-1	GCCATCAGGTTCACATCTACAGCTTCGT
GSTe6-2	CCGAGCAGGAGGGCTGCCGTCCAACTTG
GSTe6-3	TGTCATGGCATATCTTAACTGACTGGAG
GSTe6-4	CAGTCATGGATGGGTTCATACCAAGCGT
GSTe14-1	CTTCTCGAATGGCACTTCAAAGATCTCA
GSTe14-2	GCTCGTGACGGCGGACTATAGTCCATAG
GSTo2-1	GAAGTTTCTCTTGTTTGTTCCAACCATC
GSTo2-2	CCTATCGTCTGTACTCCTTGAAGTGAGG
GSTe1-2	CATCATGCACGCACGCACAGGCGGACTC
GSTe1-1	AATTCTGGAGTAAGATTTTCACCAGCCT
CarE1-F	ACCAAAATCCGACCTTTTATCACA
CarE1-R	TTTCATTGTGAATAAAATTTTAAA
CarE2-F	AGTTACTGAGTTACTATAATACCT
CarE2-R	TGTAGTTCATGGTAGTGCGTGGAG
CarE3-F	AAACGAACTAAAGAAGTAAAACAG
CarE3-R	TTGCTATGAAAAAATAAACAAGTT
CarE4-F	ACAATACTAAAAATTAAGAACAAG
CarE4-R	TGGCTGCGTCTGCTCACTGAGTTA
CarE5-F	GTATTACCTGATGGTAAGCAATCG
CarE5-R	TTGTAGATCAGCGAAGTCGGACGC
ABCB1-F	TCATTTTAAAAGACCTTTCCATTG
ABCB1-R	TATTTCACCATAGCCTCTAGTAAG
ABCB2-F	CCTTGGCTTATCAACACATACGAC
ABCB2-R	ACAGTGATGCAGGCGAGTGCCAG
ABCC1-F	TAAAGTATCTATGTAAAAAAACAT
ABCC1-R	CATGGTCGAGCCTGGCTCCCAGTA
ABCC2-F	CGATGTTTTATAGCCTATATCATT
ABCC2-R	AGGACTATTTAATATTTAGTTTGC

ABCC3-F	TTCCAGAGAAGGATGCGGCATATA
ABCC3-R	TGAATCACGATCGGTACGCTCCAG

Supplementary Table 4 The primers used for reporter and promoter constructs

Gene	Primer sequences (5'-3')
Maf-F	TCGAATTTAAAGCTTGGTACCATGCCTCATGATTTAAAGGA
Maf-R	TCGAACCGCGGGCCCTCTAGACTATGGCTGTATTTCCAATT
CncC-F	TCGAATTTAAAGCTTGGTACCATGTTTGAAGAGGAATTGGTTCTG
CncC-R	TCGAACCGCGGGCCCTCTAGATCACTGGTCGTAGCTCTTGGCTTT
CYP321B1-F	GCGTGCTAGCCCGGGCTCGAGCTCGATGATAGAGATTGGGT
CYP321B1-R	CAGTACCGGAATGCCAAGCTTGTTTGGTTGTCTGAACTTGT
CYP321A9-F	GCGTGCTAGCCCGGGCTCGAGGCTTGTTCTTCTCCATTGGAATCT
CYP321A9-R	CAGTACCGGAATGCCAAGCTTCGTTGTGTATTTTTCTGAAGCGTG
CYP321A8-core-F	GCGTGCTAGCCCGGGCTCGAGCAGTATACTGTTTCTGGGCT
CYP321A8-cncc-F	GCGTGCTAGCCCGGGCTCGAGATTTGTTAACCAAAGTCATGACTC
CYP321A8-R	CAGTACCGGAATGCCAAGCTTGTCTCTAATATTAATAAAAAATCAC

Supplementary Table 5 Binding sequences of CncC/Maf, AhR/Arnt, EcR and PXR

Gene name	CncC/Maf binding sequence	AhR/Arnt binding sequence	EcR binding sequence	PXR binding sequence
321A8	CATGACTCAGCAAAA (-196 to -182)		TAGTACATTGACCTC (-643 to -629), GAGTTATATGTACTT (-1189 to -1175)	
321A9	ATGACTGTGCAGAC (-155 to -142), ATGATTTGGCTTTT (-1055 to -1042)		GATTTCTTTGACCTT (-721 to -707)	TCTAAGTTCAA (-577 to -567)
321B1	AATGACAACGCAAAA (-237 to -223), AATGATTAAACAATA (-732 to -718)	GCAAGCGTG (-1080 to -1072)		GACAAGTTCAG (-51 to -41)
321A16	GATGACAATACAACA (-245 to -231)	GGACACGC (-647 to -640)		
9A11	ATGATTACGAAATT (-552 to -539)	ACGAGCGTG (-934 to -926)		
9A27	ATGGCTCTCCGATA (-575 to -562)	CACGCAGGG (-802 to -794)	ATGTTTGTTGACCTT (-403 to -389)	
GSTe2	CGTGAGGAGGAGGAT (-205 to -191)	CACGCCCGC (-990 to -982)	CAGTTAGTTGTCGTA (-691 to -677)	
GSTe6	AATGACAAGGCAAAA (-259 to -245)	CACGCGATG (-114 to -106)	AAGTTAATTGCATTC (-630 to -616)	CTGAACTTAAA (-226 to -216)
GSTe14	GTGGTGCAACGTAA (-890 to -877)			GTGAACTGACC (-777 to -767)
GSTo2	GATTAAACAGCAAAA (-210 to -196), GATGGCTCGTCAATA (-357 to -343)	CACGCTACG (-823 to -815)		
CarE1	AGTCATTTTGCAAAA (-1045 to -1031)		GAGTTCATTGCCATT (-76 to -62), AAATAAATTGCACTT (-972 to -958)	AATTAGTTCAC (-1023 to -1013)
CarE2		GGAGGCGTG (-970 to -962)	CACTACCATGAACTA (-15 to -1)	
CarE4		CTCAGCGTG (-309 to -301), TGCTGCGTG (-813 to -805)	AAGTACAATGCTTTA (-676 to -662)	
CarE5	ATGCTTCGGCGATA (-301 to -287), ATGATTTTTCAATA (-709 to -696)	CACGCAACG (-1107 to -1099)	GACTTCGCTGATCTA (-16 to -2)	CTCCAGTTCAG (-914 to -904)
ABCB1		CACGCTATT (-40 to -32)	AAATTAATTCCACTT (-356 to -342), ACATTTATTGAAATG (-497 to -483)	TTTCAGTTCAG (-132 to -122)
ABCB2	ATGATTCAAAAATA (-306 to -293), AATGAGTCGTAAGTA (-734 to -720)		AAGTTCACTCACTTA (-961 to -947)	AATGAGTTCAA (-426 to -416)
ABCC1		CACGCGAAT (-502 to -494)	GTATTTGTTGCCTTC (-460 to -446)	
ABCC2	AATGACGTGTTAGAT (-905 to -891)	CATCGCGTG (-752 to -744)		
ABCC3			CACTTAACTGAAACC (-172 to -158)	AACACGTTCAT (-1134 to -1124)

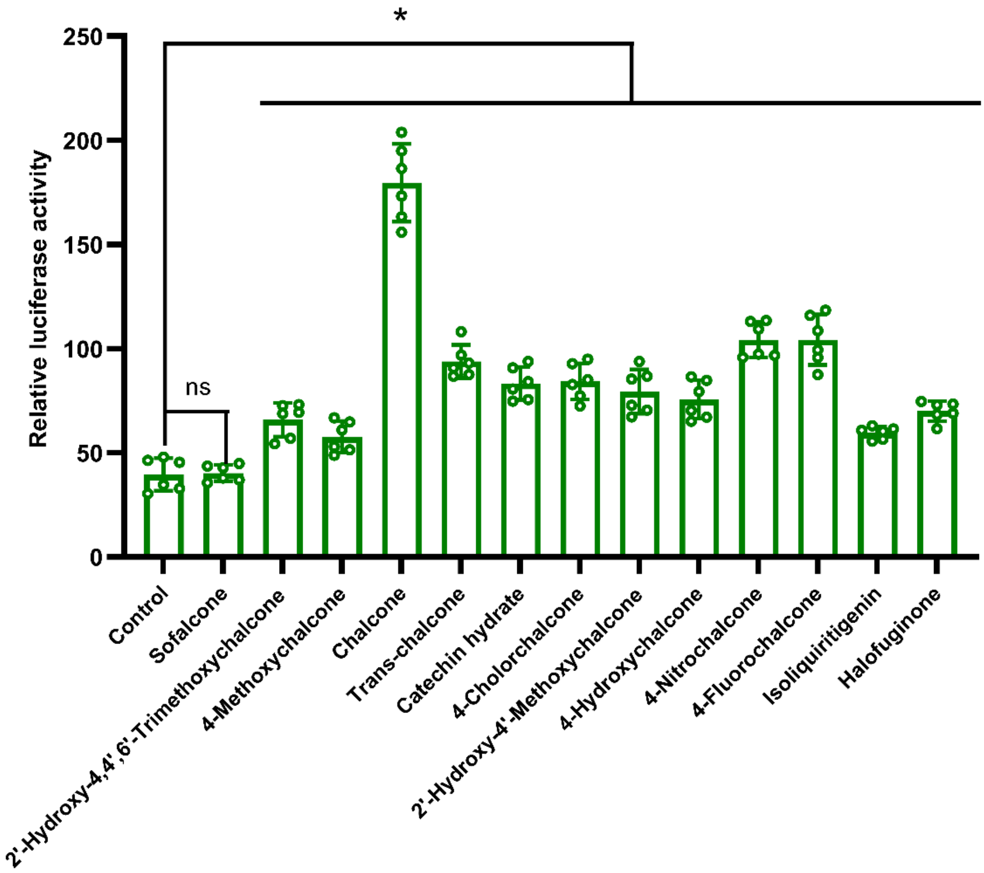

Supplementary Figure 1. The screening of inhibitors of *CncC/Maf* in sf9 cells. The PGL3-core-CncC promoter construct was transfected into sf9 cells. After 16h post-transfection, natural compounds (at a final concentration of 2.5 μM) or an equal volume of DMSO (control) was added. After 48h transfection, the cells were collected and the luciferase activity was measured. n = 6.

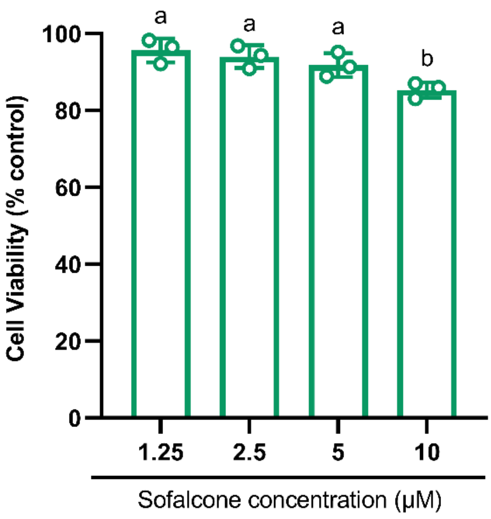

Supplementary Figure 2. Effect of sofalone on the viability of sf9 cells. The sf9 cells (2×10^5 cells/mL) were

seeded in 96-well culture plates and treated in triplicates with increasing concentrations for sofalone, and the cell viability was estimated using an MTT assay. The results are presented as the mean ± SD. Student's t-test was used for statistical analysis. Letters a and b denote significant differences at $p < 0.05$. n = 3.

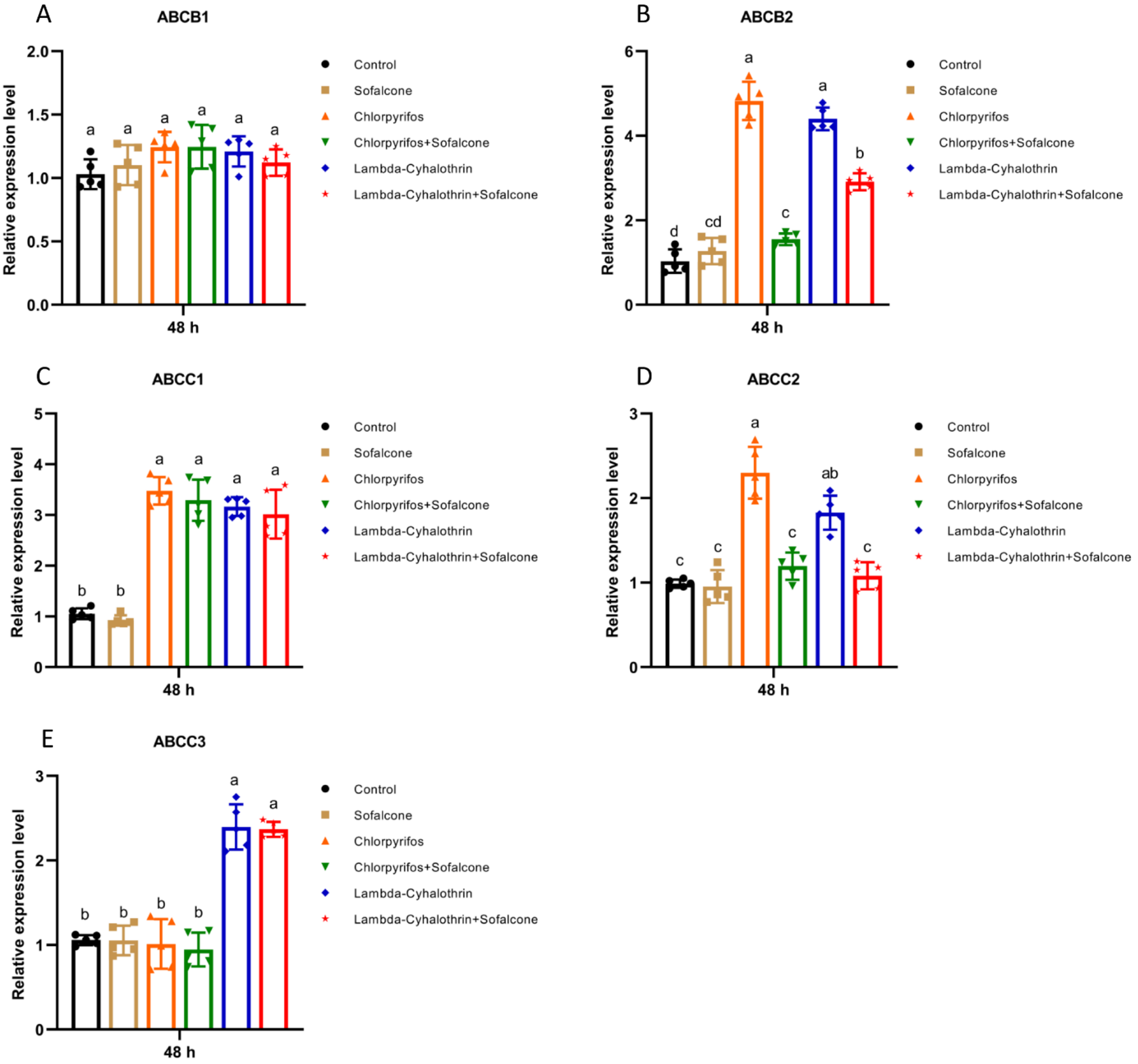

Supplementary Figure 3. Sofalcone inhibited the transcription levels of ABC genes under the stress of insecticides. The expression difference of ABC genes in response to insecticides with or without sofalcone was analyzed in three biological replicates, each with three technical replicates, by quantitative real-time PCR. The results are presented as the mean ± SD. Different letters on the error bars indicate significant differences based on ANOVA with Tukey's HSD multiple comparison test ($p < 0.05$). n = 5.

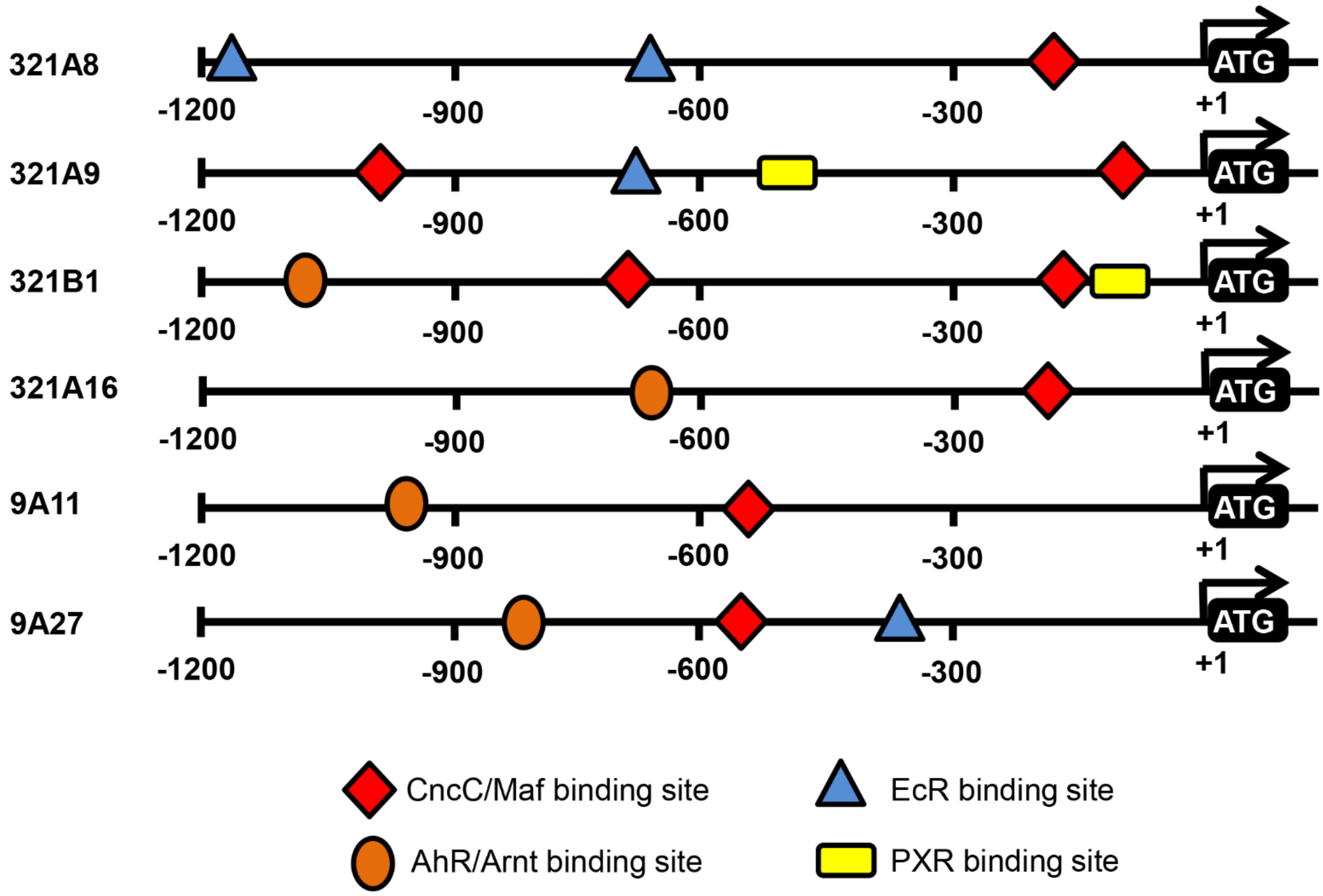

Supplementary Figure 4. Prediction of transcription factor binding sites in the promoter regions of P450 genes. The nucleotides are numbered relative to the translation start site (ATG) indicated by +1, with sequence upstream of it preceded by '–'. Search was focused on the presence of the binding sites of *CncC/Maf*, *AhR/Arnt*, *ECR* and *PXR*.

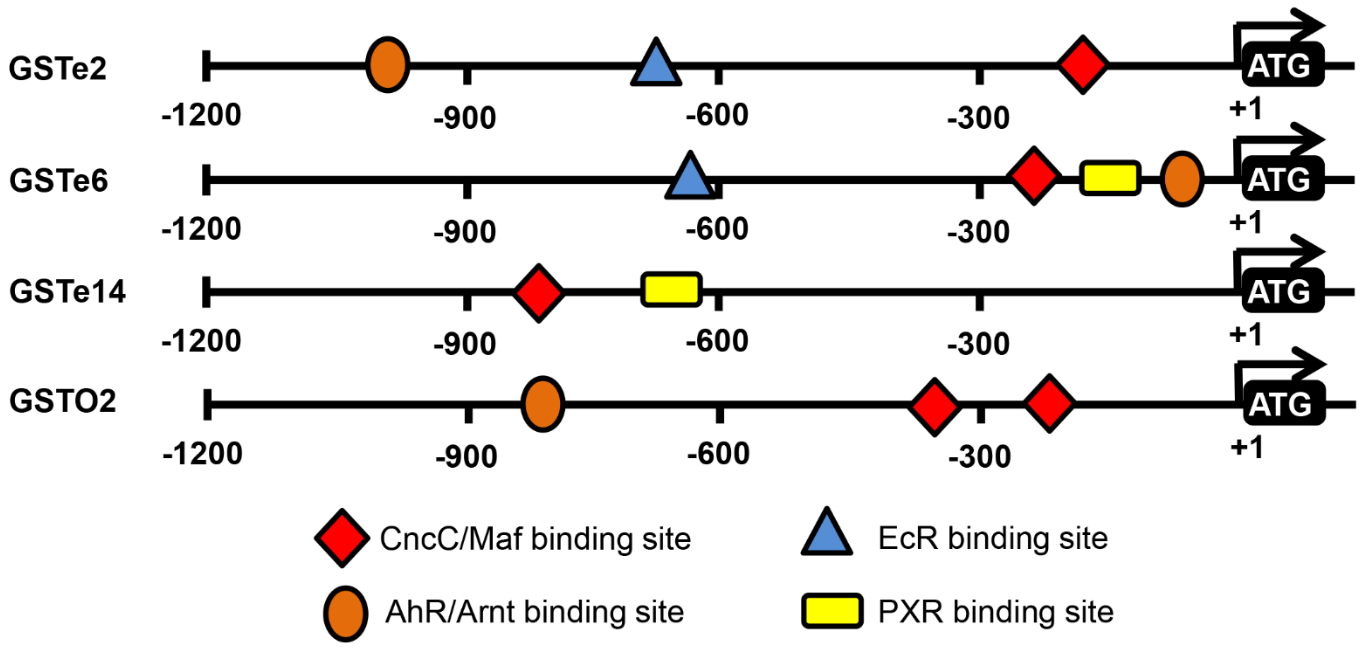

Supplementary Figure 5. Prediction analysis of the promoter regions of GST genes. The nucleotides are numbered relative to the translation start site (ATG) indicated by +1, with sequence upstream of it preceded by "-". The analysis was performed with JASPAR and ALLGEN software. The transcription factor binding sites are shown in different colors.

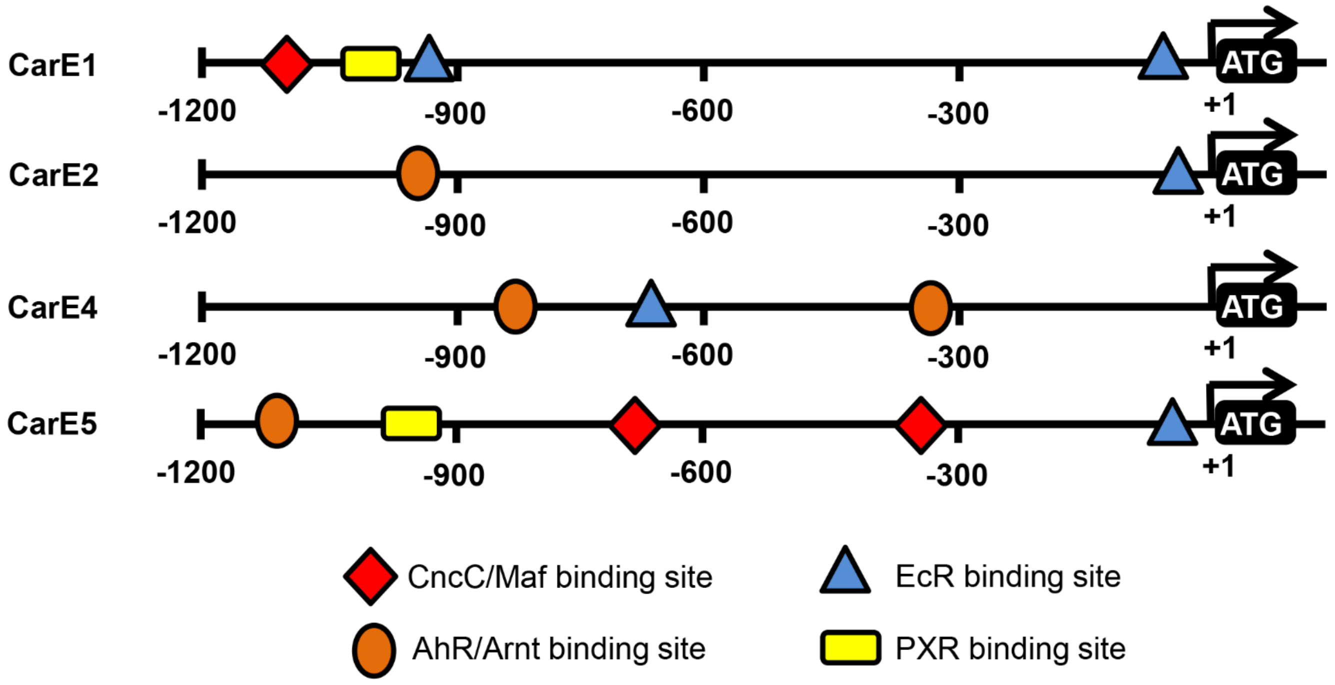

Supplementary Figure 6. Prediction of TF binding sites in the promoter sequences of CarE genes. The nucleotides are numbered relative to the translation start site (ATG), with sequence upstream of it preceded by "-

“. Four different colors represent the binding sites of *CncC/Maf*, *AhR/Arnt*, *ECR* and *PXR*, respectively.

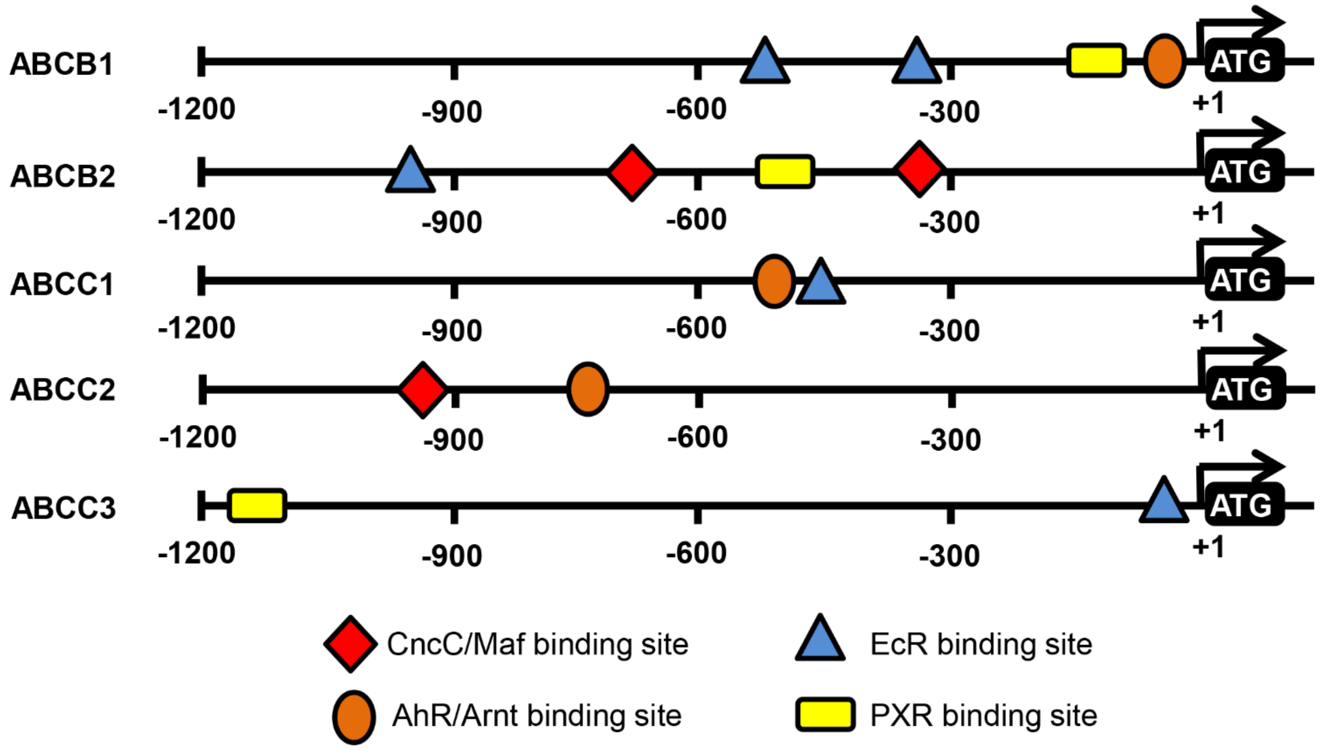

Supplementary Figure 7. Prediction of *CncC/Maf*, *AhR/Arnt*, *ECR* and *PXR* in the promoter region of ABC genes. The position denotes the start codon ATG and putative binding sites for transcription factors are indicated in different colored boxes. The binding sites were evaluated in silico by the JASPAR and ALLGEN software.

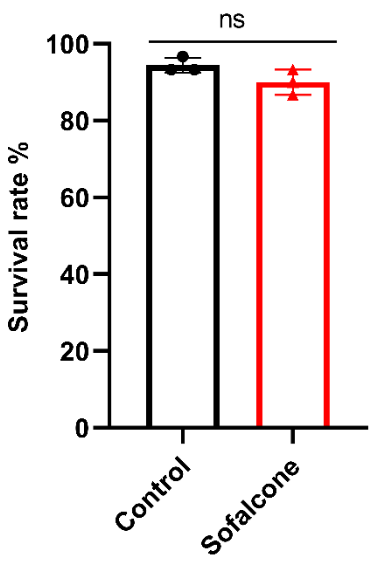

Supplementary Figure 8. The survival rate of *S. exigua* in response to sofalcone for 72h. Data are the

mean ± SD of three independent assays. n = 3.

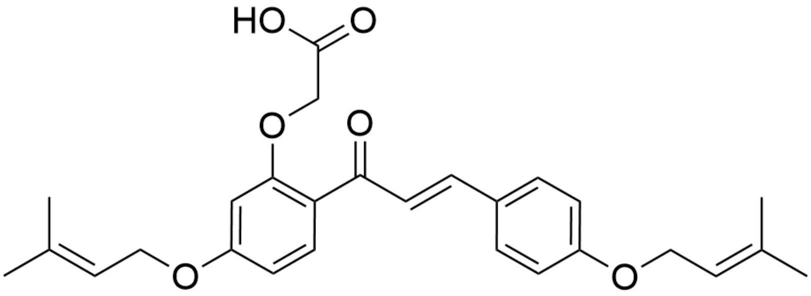

Supplementary Figure 9. The chemical structure of sofalcone.
