# Supplementary material for: Inhibition of transcriptional regulation of detoxification genes contributes to insecticide resistance management in Spodoptera exigua
Source: Commun Biol. 2025 Jan 27;8:128. doi: 10.1038/s42003-025-07560-8 (PMC11772755; doi:10.1038/s42003-025-07560-8)
Supplement: Supplementary file 3 — Reporting Summary [file 42003_2025_7560_MOESM3_ESM.pdf]

nature portfolio

Corresponding author(s): Jianya Su

Last updated by author(s): Oct 25, 2024

Reporting Summary

Nature Portfolio wishes to improve the reproducibility of the work that we publish. This form provides structure for consistency and transparency in reporting. For further information on Nature Portfolio policies, see our Editorial Policies and the Editorial Policy Checklist.

Statistics

For all statistical analyses, confirm that the following items are present in the figure legend, table legend, main text, or Methods section.

n/a	Confirmed	
☐	☒	The exact sample size (*n*) for each experimental group/condition, given as a discrete number and unit of measurement
☐	☒	A statement on whether measurements were taken from distinct samples or whether the same sample was measured repeatedly
☐	☒	The statistical test(s) used AND whether they are one- or two-sided<br>*Only common tests should be described solely by name; describe more complex techniques in the Methods section.*
☒	☐	A description of all covariates tested
☐	☒	A description of any assumptions or corrections, such as tests of normality and adjustment for multiple comparisons
☐	☒	A full description of the statistical parameters including central tendency (e.g. means) or other basic estimates (e.g. regression coefficient) AND variation (e.g. standard deviation) or associated estimates of uncertainty (e.g. confidence intervals)
☐	☒	For null hypothesis testing, the test statistic (e.g. *F*, *t*, *r*) with confidence intervals, effect sizes, degrees of freedom and *P* value noted<br>*Give P values as exact values whenever suitable.*
☒	☐	For Bayesian analysis, information on the choice of priors and Markov chain Monte Carlo settings
☒	☐	For hierarchical and complex designs, identification of the appropriate level for tests and full reporting of outcomes
☒	☐	Estimates of effect sizes (e.g. Cohen's *d*, Pearson's *r*), indicating how they were calculated

Our web collection on statistics for biologists contains articles on many of the points above.

Software and code

Policy information about availability of computer code

Data collection	GraphPad Prism 8.0, PoloPlus and Excel
Data analysis	GraphPad Prism 8.0, PoloPlus and Excel

For manuscripts utilizing custom algorithms or software that are central to the research but not yet described in published literature, software must be made available to editors and reviewers. We strongly encourage code deposition in a community repository (e.g. GitHub). See the Nature Portfolio guidelines for submitting code & software for further information.

Data

Policy information about availability of data

All manuscripts must include a data availability statement. This statement should provide the following information, where applicable:

- Accession codes, unique identifiers, or web links for publicly available datasets
- A description of any restrictions on data availability
- For clinical datasets or third party data, please ensure that the statement adheres to our policy

All data generated or analyzed during this study are included in the manuscript and Supplementary material. Supplementary material contains all primers used to amplify gene sequences and construct expression vectors. All the sequences have been deposited in GenBank (Accession nos: MK275645- MK275647).

Human research participants

Policy information about studies involving human research participants and Sex and Gender in Research.

Reporting on sex and gender	n/a
Population characteristics	n/a
Recruitment	n/a
Ethics oversight	n/a

Note that full information on the approval of the study protocol must also be provided in the manuscript.

Field-specific reporting

Please select the one below that is the best fit for your research. If you are not sure, read the appropriate sections before making your selection.

☒ Life sciences ☐ Behavioural & social sciences ☐ Ecological, evolutionary & environmental sciences

For a reference copy of the document with all sections, see nature.com/documents/nr-reporting-summary-flat.pdf

Life sciences study design

All studies must disclose on these points even when the disclosure is negative.

Sample size	At least three biological replicates are used, and the sample size details are described in the figure legends
Data exclusions	There are no specific data exclusions for data analyses
Replication	Most of findings from this study in main text were all confirmed by another independent experiment
Randomization	Animal study are randomized
Blinding	No relevant because all the bioassay or in vitro experiments performed in this study do not require blinding of the subjects.

Reporting for specific materials, systems and methods

We require information from authors about some types of materials, experimental systems and methods used in many studies. Here, indicate whether each material, system or method listed is relevant to your study. If you are not sure if a list item applies to your research, read the appropriate section before selecting a response.

Materials & experimental systems

n/a	Involved in the study	
☒	☐	Antibodies
☐	☒	Eukaryotic cell lines
☒	☐	Palaeontology and archaeology
☐	☒	Animals and other organisms
☒	☐	Clinical data
☒	☐	Dual use research of concern

Methods

n/a	Involved in the study	
☒	☐	ChIP-seq
☒	☐	Flow cytometry
☒	☐	MRI-based neuroimaging

Eukaryotic cell lines

Policy information about cell lines and Sex and Gender in Research

Cell line source(s)	Sf9 cell lines. This cell lines was developed from ovary of Spodoptera frugiperda.
Authentication	The cells were authenticated by PCR amplification of selected genes.
Mycoplasma contamination	Cells were not tested for mycoplasma contamination

Commonly misidentified lines (See ICLAC register)	n/a

Animals and other research organisms

Policy information about studies involving animals; ARRIVE guidelines recommended for reporting animal research, and Sex and Gender in Research

Laboratory animals	The susceptible strain of S. exigua used in this study was obtained from Wuhan Kernel Bio-pesticide Company, Hubei, China. The resistant strain of S. exigua was collected in 2016 from Welsh Onion, Allium fistulosum, in Huizhou, Guangdong province, China. Larvae were reared on an artificial diet at 25 °C under a 16-h light/8-h dark photoperiod with a relative humidity of 60 ± 5%
Wild animals	Not Applicable
Reporting on sex	Not Applicable
Field-collected samples	Not Applicable
Ethics oversight	Not Applicable

Note that full information on the approval of the study protocol must also be provided in the manuscript.
